# Tools for Discussing Identity and Privilege Among Medical Students, Trainees, and Faculty

**DOI:** 10.15766/mep_2374-8265.10864

**Published:** 2019-12-20

**Authors:** Candace J. Chow, Gretchen A. Case, Cheryl E. Matias

**Affiliations:** 1 Associate Director of Education Research, Office of Curriculum, University of Utah School of Medicine; 2 Chief, Program in Medical Ethics and Humanities, Division of General Internal Medicine, Department of Internal Medicine, University of Utah School of Medicine; 3 Associate Professor, Division of General Internal Medicine, Department of Internal Medicine, University of Utah School of Medicine; 4 Associate Professor, School of Education and Human Development, University of Colorado Denver

**Keywords:** Social Identity, Social Identification, Identity Awareness, Privilege, Health Equity, Bias, Culturally Responsive Care, Diversity, Inclusion

## Abstract

**Introduction:**

Physicians and students of all backgrounds should be prepared to interact with patients of various socioeconomic, racial, ethnic, gender, religious, and sexual orientation identities. The approach described here emphasizes how important it is for physicians and physicians-in-training to develop self-awareness before engaging with patients.

**Methods:**

Over the course of 6 months, we conducted workshops on identity awareness for four groups: (1) fourth-year medical students (*N* = 6), (2) first-year medical students (*N* = 88), (3) faculty and staff (*N* = 11), and (4) residents/fellows (*N* = 4). Exercises in this workshop prompted learners to reflect on the development of social and professional identities through the use of an identity wheel activity, a group reading about professional identity formation, and a hands-on activity modeling social inequity.

**Results:**

Our analysis of responses to pre- and postsurveys indicated that learners in the first-year medical student group (*N* = 88) experienced increased awareness and acknowledgment of social identity, professional identity, professional relationships, and the concepts of privilege and difference following participation in this workshop.

**Discussion:**

These exercises guide learners toward critical thinking about privilege and identity to better prepare them for culturally inclusive patient interactions. These materials can be used with physicians at various levels of training. The earlier they are used, the more time learners will have to reflect on social and professional identities before interacting with patients.

## Educational Objectives

By the end of this activity, learners will be able to:

1. Name the identities that matter in their personal lives and professional careers.
2. Describe how their personal identities are connected to identity group memberships.
3. Explain how their personal identities are connected to professional roles.
4. Discuss how social identities are embedded within social structures.

## Introduction

Today's physicians work with an increasingly diverse patient population in an environment where diversity of representation is often celebrated but systemic discrimination is ignored. One way to minimize implicit and explicit discrimination in health care settings is to prepare physicians of all backgrounds to interact with patients of various class,^1^ racial, ethnic, gender, religious, and sexual orientation identities. The tools described here equip faculty to help students become aware of identities and the social structures that result in systemic inequity.

Undergraduate medical education teaches future physicians how to be physicians.^2–5^ A major critique of undergraduate medical cross-cultural education is that it emphasizes cultural competence—knowledge about different social groups and how to work with them—rather than emphasizing self-awareness.^6^ Courses do not often, if at all, address issues of power and privilege.^7,8^ In turn, because students are not taught how to be critically conscious of privilege with regard to patient interactions,^9^ they do not understand how diversity education is relevant to their practice.^8^ Previously published works in *MedEdPORTAL* provide guidelines on how to teach cultural competence^10–12^ and social determinants of health.^1,13^ They use case studies and multimedia to teach students how to take more culturally sensitive approaches to interviewing and diagnosing patients.^14–16^ Several resources utilize self-reflection in their approach to learning cultural competence.^17–19^

This workshop focuses on introspection and privilege. We move beyond a cultural competence approach^5^ by inviting learners to reflect on their own identities before learning about social determinants of health and how to conduct patient interviews. This approach is informed by literature on how “critical analysis and understanding of difference and dominance”^20^ are key to understanding how social inequities stem from differences in privilege and power.^9^

Below, we describe three activities (detailed in the appendices). Although none are original creations, they have been adapted from use in other disciplines (higher education and teacher education) and combined to meet specific learning goals. Over a period of 6 months, we conducted four workshops that used the activities in different ways and combinations. Two of the workshops were conducted during didactic sessions, and two were extracurricular. The first workshop was one of 10 sessions in a medical humanities elective course for advanced medical students. The second workshop was an optional session in a weeklong transition to medical school program. The third workshop was attended by faculty and staff from across the health sciences at our institution's education development symposium. The fourth workshop was held for trainees at the request of one of the faculty who attended the symposium and was incorporated into the weekly didactic sessions required of endocrinology fellows and residents.

## Methods

The activities (Table 1) helped learners explore the multiple facets of their identities and recognize how their identities were connected to a system perpetuating inequity. They could be used alone or in combination with each other. We encourage others to use the activities in the arrangement that best suits their desired educational objectives and available time.

**Table 1.** Overview of Exercises

Exercise	Objectives Covered	Educational Format	Time	Learners
Ground rules	N/A	Facilitated discussion	10 minutes	Used with all four groups (fourth-year medical students, first-year medical students, faculty, and residents/fellows)
Identity wheel	1. Name the identities that matter in learners’ personal lives and professional careers. 2. Describe how personal identities are connected to identity group memberships. 3. Explore how personal identities are connected to professional roles. 4. Discuss how social identities are embedded within social structures.	Independent work, small-group discussion, and facilitated large-group discussion	40+ minutes	Used with all four groups (fourth-year medical students, first-year medical students, faculty, and residents/fellows)
Group reading	3. Explore how personal identities are connected to professional roles.	Facilitated large-group reading and discussion	20–50 minutes (depending on how the reading is done)	Used with two groups (fourth-year medical students, first-year medical students)
Marshmallows and pretzels	4. Discuss how social identities are embedded within social structures.	Small-group work and facilitated large-group discussion	30–50 minutes (depending on time available for discussion)	Used with three groups (first-year medical students, faculty, and residents/fellows)

### Ground Rules

Prior to doing any of these activities, we worked with learners to establish mutual expectations through shared ground rules. Ours included the following: (1) Be an active listener, (2) use “I” statements, (3) share airtime, (4) be respectful of others’ opinions, (5) respect confidentiality, and (6) courageously ask questions. We elicited suggestions from learners and supplemented them if any key rules were missing (e.g., confidentiality, respect, active listening). Establishing ground rules at the beginning made it easier to address violations of the rules if they arose.

### Identity Wheel

This activity (Appendices A and B) was aligned with Educational Objectives 1–4 and took at least 40 minutes to complete. It was intended to help learners reflect on their social identities and think about how their identities were embedded within a hierarchical social structure. Learners completed graphic organizers, colloquially called *identity wheels*, that contained two concentric circles. The inner circle was labeled “given” and contained descriptors of identities with which people were born (e.g., age, nationality, sex, race). The outer circle was labeled “chosen” and contained descriptors of identities learners had chosen for themselves (e.g., geographic location, religion, marital status, career). We acknowledged that given identities could be fluid (e.g., gender, nationality) and that not everyone had the agency to choose their chosen identities (e.g., geographic location). Learners filled out the wheels independently and then shared (in pairs or small groups) initial reactions to the activity. Finally, we led a facilitated large-group discussion about what it was like to complete the wheel, which identities participants wrote in each section, which identities were most salient, and from which identities they benefited. We also asked the group to discuss how these identities facilitated or hindered the work they did as professionals. Finally, we closed the session by discussing how personal identities and professional identities informed one another.

### Group Reading

This activity (Appendix C) was aligned with Educational Objective 3 and took at least 20 minutes. It was used to get learners to think about the development of a professional identity. The Clandinin, Cave, and Cave article^21^ provided students with an example of how one physician-in-training went from feeling like an imposter physician to feeling comfortable in her role. We recommend reading the article aloud together to clarify points along the way, especially if this reading is done with first- and second-year medical students, who may be less familiar with the terminology used in the article. If a group is not able to read the entire article together, Table 1 in Clandinin et al. (“Leslie's Final Parallel Chart”)^21^ offers a brief, clear summary of these issues. After reading the article, we led learners in a discussion about the value of parallel charts, the perils of imposter syndrome, and how learners had recognized or might recognize moments of transition in their own professional identity. We also asked learners to consider how their personal identities intersected with their professional identities. If the learners were early in their career, we asked them to think about what facets of personal identity they wanted to bridge with their professional identity and career. We concluded by asking learners to think about and write down three things they would like to see reflected in their professional identity in the next 5 years.

### Marshmallows and Pretzels

This activity was aligned with Educational Objective 4 and took at least 30 minutes to complete. The marshmallow and pretzel exercise (Appendix D), which has been used in teacher education and higher education settings, was intended to teach learners about inequity and privilege by demonstrating how resources were distributed inequitably among different groups of people. It also demonstrated that simply leveling the playing field or otherwise trying to equalize resources would not completely solve this problem. Learners were asked to split into small groups, ranging from two to five people. Each small group was given a brown paper bag, which contained a random combination of large and small marshmallows and large and small pretzels. At least one group received only marshmallows or only pretzels. Groups were asked to build the tallest tower possible using only the marshmallows and pretzels. Because groups did not have the same materials, the towers they were able to build varied greatly in height and stability. Once everyone had had a chance to build the towers, we asked learners to pause and look around the room. We led a short large-group discussion about what they noticed about the size of the towers, how their towers and materials compared to those of others, and how this exercise was applicable to real life. We concluded this discussion by admitting that we had rigged the system and that we now wanted to level the playing field by giving everyone equal numbers and sizes of pretzels and marshmallows and a chance to build again. However, before we started the second round of building, we told learners that they were allowed to build on top of their existing structure. We gave out the new materials and invited the groups to resume building. After a few minutes, we stopped the building and engaged learners in another large-group discussion. This time, we asked them why leveling the playing field was not a sufficient way to address historic and systemic inequity. We also asked learners to reflect on how privilege was something that needed to be continually addressed and how they could use their privilege as physicians or physicians-to-be in the future. We closed by reflecting on how all of the activities tied together and asked learners how they could be more conscious of their privilege and the concept of privilege in their medical practice.

### Evaluation

We presented this session to four different groups: (1) fourth-year medical students in an elective course (*N* = 6), (2) first-year medical students during an optional orientation training (*N* = 88 of 128 students), (3) faculty and teaching staff attending a health science educators symposium (*N* = 11), and (4) residents and fellows during a didactic session (*N* = 4). To measure whether educational objectives were met, we adapted an existing, previously validated scale^22^ measuring collective self-esteem to conduct pre-/postsurveys (Appendix E). We used these items to ask learners how their social group memberships influenced personal and professional identities. The questions used a 7-point Likert scale (1 = *strongly disagree*, 7 = *strongly agree*) and included items such as “The social groups I belong to are unimportant to my sense of what kind of person I am.” We expected that learners, after participating in the workshop, would more strongly agree that social groups overlapped with personal and professional identities because they would have a better sense of the social groups to which they belonged.

## Results

We were granted an exemption by our Institutional Review Board to collect and analyze data. We used a two-sample *t* test to assess whether there were any significant changes on these measures between the pre- and postsurveys. Only one group we worked with was large enough (*N* = 88) to produce meaningful survey results, so we present survey data only from group 2 (first-year medical students). We report means for each of the survey questions and indicate which findings are statistically significant at the *p* < .05 level using a one-tailed test (Table 2).

**Table 2.** First-Year Medical Students’ Responses to Identity Scale (*N* = 88)

Question	Presurvey	Postsurvey
Overall, my group memberships have very little to do with how I feel about myself.	3.8	3.5
The social groups I belong to are an important reflection of who I am.	5.3	5.3^a^
The social groups I belong to are unimportant to my sense of what kind of person I am.	3.0	3.1
In general, belonging to social groups is an important part of my self-image.	4.5	4.9
The social groups I belong to have influenced my decision to become a physician.	3.7	4.2^b^
Overall, my group memberships have very little to do with how I feel about practicing medicine.	4.1	4.0

a *p* = .045.

b *p* = .004.

The quantitative findings suggest some differences in how the group 2 workshop learners thought about their identities after the exercises. Changes in means are statistically significant for two questions: “The social groups I belong to are an important reflection of who I am” and “The social groups I belong to have influenced my decision to become a physician.” These data reveal that group 2 learners were more likely to agree that their social groups reflected who they were after participating in the workshop. Additionally, they were more likely to agree that their social identities were connected to their decisions to pursue medicine after completing the workshop.

The open-ended comments provide evidence that this workshop and its activities are useful in promoting thought and reflection around social identities, professional identities, the intersection of social and professional identities, how identities are socially constructed, and how one's identity intersects with one's social location and privilege.

To assess what stood out most significantly to learners from these exercises, we asked an open-ended question of all groups: “Thinking back over today's session, was there any particular concept that resonated with you? Did you have an ‘ah-ha’ moment? If so, what was it about?” Our goal in asking this question was to learn which components respondents found useful and important. Learners’ responses were coded into four themes: (1) awareness of social identity, (2) awareness of professional identity, (3) recognition of professional relationships, and (4) acknowledgment of privilege and difference.

### Theme 1: Awareness of Social Identity

Learners who indicated that they had learned something related to social identity wrote about acknowledging an identity for the first time, learning that their social identities affect how they are seen by others, and realizing that some identities are more salient than others. For example, one respondent stated, “I appreciated our discussion about evolving one's identity and the fluidity involved in how we distinguish ourselves.” Another respondent wrote, “The parts of your identity that you think about most often are the parts different [from] the majority [population], and things that are visible/[that you're] unable to hide.”

### Theme 2: Awareness of Professional Identity

Learners wrote about how the workshop influenced their thinking regarding professional identity and how identity development is an ongoing process. Sample responses included “I think that the moment when we ‘become doctors’ would not be one single moment but instead it will be a slow transition. Each of those pieces and moments will make us the doctors we will become in the future,” and “Becoming a physician is full of ‘fake it til you make it’ experiences.”

### Theme 3: Recognition of Professional Relationships

Some learners wrote about how the workshop helped them think about working with others and the characteristics needed in a team setting. One respondent wrote, “Sometimes being a good leader means taking a step back and letting someone with more knowledge handle the situation.” Another participant wrote, “Teamwork in medicine is super important.”

### Theme 4: Acknowledgment of Privilege and Difference

Many learners explained how the workshop made them think about privilege and difference in some way. One participant shared, “I just did not quite understand how hard it is to make things equal and fair.” Another participant wrote, “I thought about how racism still plagues our society and things we can do to change it.” A third participant noted, “We can breakdown assumptions to become better providers.”

## Discussion

These activities employ active learning to teach about personal and professional identities. We conducted four workshops by using various combinations of these activities depending on the time allotted, the size of the group, and the goals for the learners. The data suggest that these activities are useful tools for discussing identity awareness and privilege among physicians and physicians-in-training. The survey data indicate that asking learners to identify their social groups is related to how they think about social group membership. The open-ended comments indicate that these exercises are impactful in raising awareness of identities, professional relationships, and privilege. We also received informal positive feedback from individuals who approached us at the end of the workshops, including one faculty member who invited us to conduct a workshop with her trainees.

Implementing the exercises in different settings taught us that setting a tone allowing for hard questions and difficult conversations was important. Setting ground rules was a helpful way to do this. Trying the activities with different groups also reinforced that although each activity was self-contained and could be used in any combination or order (we customized lesson plans for each workshop), there was a flow that worked best. The identity wheel was most effective for beginning the discussion because it introduced the topic of identities and provided common language for discussion. The marshmallow and pretzel exercise was best used later in the session, once context had been established and we had discussed how different group memberships afforded us different opportunities. These activities can be used with clinicians at various levels of training. However, some people may find discussions of identity to be sensitive and have concerns about sharing personal information with supervisors or perceived superiors. Thus, we recommend that these activities be used among peers rather than in mixed settings (e.g., in an educators-only training session rather than in a mixed group of students and educators).

Challenges to this work included time constraints, level of priority, and affronts to our personal identities. Each of the three exercises can be accomplished within 30–40 minutes but is enhanced by additional time for in-depth discussion. We felt pressed for time during our shorter sessions. Due to high demands on medical student contact hours at our own institution, we have only been able to offer these activities in optional, cocurricular settings. During the second session, Candace J. Chow was questioned about her use of identifiers by a student. She used it as an opportunity to discuss microaggressions and institutional racism but needed to debrief the encounter with us after the workshop as a way to process the racial microaggression that the student had inflicted on her. Facilitators who use these exercises should be comfortable talking about their own life narratives and experiences with discrimination and identity privilege. We offer guiding questions and annotations of optional readings in the appendices for those who are less familiar with these concepts.

Our results are not generalizable, due to small sample sizes, the nature of qualitative data, and the fact that we have taught this material with a heterogeneous group of learners (students, trainees, and educators). Yet the fact that we have successfully taught this material to different populations shows how important it is to present these topics to all learners. If not strictly generalizable, the material certainly can be modified according to resources and needs. Another limitation to our work includes the cocurricular nature of the sessions. Despite not being able to include these activities in the required curriculum, we felt it was important to cover them during available opportunities (in our case, optional and mostly cocurricular). Incorporating the various activities into the curriculum at different time points (e.g., identity wheel at the beginning of the first semester, group reading at the beginning of the second semester) could be one way to deliver this content in a sustained and iterative fashion so that students do not see this as a onetime thing but as an important touch point throughout their education.

Feedback from learners included requests for strategies or techniques that they could implement in the clinic, based on awareness of their own identities. In the future, we would like to better develop the connection between self-awareness of identity and medical practice. For example, future sessions could explore how one's own identity plays into decision making or how it influences interactions with patients. Future work could also examine how recognizing one's identity (and the identity of others) might translate into better care for patients.

## Appendices

A. Identity Wheel Instructions.docx
B. Identity Wheel Handouts.docx
C. Group Reading.docx
D. Marshmallow and Pretzel Activity.docx
E. Survey.docx All appendices are peer reviewed as integral parts of the Original Publication.
