# Supplementary material for: Tools for Discussing Identity and Privilege Among Medical Students, Trainees, and Faculty
Source: MedEdPORTAL. 2019 Dec 20;15:10864. doi: 10.15766/mep_2374-8265.10864 (PMC7012312; doi:10.15766/mep_2374-8265.10864)
Supplement: Supplementary file 1 — A. Identity Wheel Instructions.docx B. Identity Wheel Handouts.docx C. Group Reading.docx D. Marshmallow and Pretzel Activity.docx E. Survey.docx [file mep-15-10864-s001.zip › A. Identity Wheel Instructions.docx]

**Appendix A: Identity Wheel**

Part 1: Rationale/background

This module is designed to get participants thinking and talking about their social identities (e.g., race,

ethnicity, class, gender, sexual orientation), how these identities are socially constructed, and how these

identities influence professional identities and the professional work that we do. For many of our students, particularly those who are from majority backgrounds, it is the first time that they have been asked to think about how their experiences are reflective of the identities they embody. For some, this is also the first time they are challenged to consider that others do not experience life the way they do, and that our identities and experiences are socially constructed. This exercise has been used at various institutions (a web search for “identity wheel” returns multiple results) and the authors have completed similar exercises during various trainings. We have adapted the exercise for our purposes by including “given” and “chosen” identity descriptors on the wheel.

The identity wheel (author owned) is divided up into spaces, each of which has a different identity descriptor. When we use the wheel, we ask participants to write in identities that match with each descriptor (e.g., socioeconomic stats = middle-income). We have found it useful to differentiate between sex (e.g., female, male) and gender (e.g., woman, man, gender nonconforming), between race (e.g., Asian, Black/African American) and ethnicity (e.g., Italian, Korean), and between ethnicity and nationality (e.g., American, British, Australian) since they are often confused for one another.

The circle is designed so that the inner circle contains given identity descriptors (identities we are born with) and chosen identity descriptors (identities that we can change and choose to adopt). We acknowledge that not everyone will agree with these. For example, we list geography and religion as chosen identities, acknowledging that not everyone has the privilege to choose where to live, and that the religions we practice are often handed down to us. In addition, we list sexual orientation is as a given identity while acknowledging the complex discussions around sexual orientation and choice. While some facilitators and learners may find that the terms “chosen” and “given” oversimplify these concepts, we have found that these terms provide a good starting point for discussion. In particular, we have used these as ways to discuss how our perspectives are often socially constructed through the identities we are “born” into.

Finally, although the identities are listed separately, our questions around which identities individuals think about most or least provide opportunities to talk about when and why we find some identities more salient than others, and how identification is often contextual. Moreover, these questions allow us to discuss how identities are intersectional—none of us are singularly women, or White, or middle-class. It is the combination of these gender/race/class identities that have shaped our experiences and perspectives. Recognizing each of these identities allows us to begin examining identity and privilege, but ultimately, it is important to understand how identities work together and, at times, in tension, to inform identity.

Part 2: Individual exercise (10 minutes)

There are two different versions of the identity wheel (Appendix B). One is pre-filled with descriptors and one is blank. We use the wheel that is pre-filled, but created a blank one for those who prefer to let learners decide which descriptors to use or for those who want to fill in the wheel with their own categories.

1. Pass out the identity wheel handout.
2. Ask participants to spend 10 minutes individually filling out the identity wheel.
3. Gather participants back for a discussion

Part 3: Discussion (30 minutes+)

1. Ask learners to turn to a partner. Ask pairs to talk about what the experience of filling out the wheel was like.
   1. For how many of you was this the first time doing an exercise like this?
   2. What was this process like for you? (Was it easy or hard? How did it make you feel?)
2. Gather everyone into a large group. Ask volunteers to share what was just discussed in paired groups.
3. Continue discussion by using the following questions:

Exploring social/personal identities:

1. What are the identities you think about most often? (Instead of just asking learners to name these, we often ask learners to show, by a raise of hands, which identities they think about. We go through the list of identities and ask them to raise their hands if this is an identity they think about often.)
2. What identities do you think about least often? (As above, we often ask learners to show, rather than tell, which identities they think about least often.)
3. Which identities do you notice most about others?
4. Why is it that some of our identities are more salient than others?
5. Where do these identity categories come from?

Exploring the connection between social and professional identities:

1. Which identities give you privilege in your profession? (You may need to define privilege. A commonly accepted definition is that privilege is a special advantage that some, but not all people enjoy.)
2. Which identities do you notice most about your patients?
3. Which identities do your patients notice most about you?
4. Are there parts of your identity that are “core” to you, that do not change between settings?
5. What are some ways to bridge the differences between your identity and those of your (future) patients?

For educators (we only ask these when we are working with educators):

- 1. How similar are your identities to those of your students’?
  2. Do these differences get in the way of building relationships?
  3. What are some things we can do to acknowledge our students’ different identities/experiences?

1. Closing:
   1. Conclude the discussion with a closing statement about how our personal identities and lives influence our professional identities and lives, and vice versa, whether we are conscious of it or not.
   2. Emphasize that it is important to be aware of this back-and-forth process because self-awareness will better prepare us to interact with patients (and students, and others) who come to us with a diverse set of experiences.
   3. Remind learners that it is better for us to be thinking about these things out now, before we meet with patients, rather than to be confronted with a difficult question about identity during a patient encounter.

Part 4: Potential challenges

It is not unusual for learners to be resistant when asked to talk about issues of identity and privilege. Below we provide examples of some of the questions we have received and how we have responded.

1. What if a learner finds it hard to understand that people think about identity differently and/or that identities are socially constructed?

- We have actually found that most participants do not push back on the assertion that we all embody different social identities after going through the questions above. After doing the exercise and visually seeing how different individuals think about different identities most and least, participants seem to accept that this is different for everyone. In addition, because it’s women who usually indicate that they think about gender often, and it’s usually people of color who indicate that they think about race and ethnicity often, we are able to use that data to make the point that we are often more mindful of the identities in which they are the minority, and that people are less mindful of the identities in which they are the majority. That said, if you do get this question:
- **Where this is coming from**: A learner who finds it hard to accept that people think about identities differently has likely grown up in a very homogenous environment and has little exposure to people who are different from them. They assume that everyone (including the few minoritized individuals they’ve interacted with) must have the same experience as they have had.
- **Suggested response**: Acknowledge that there are biological explanations for differences—men and women are biologically different, as are people with different abilities. However, the reason these differences matter is because our societal system is set up so that some people (e.g., men, straight individuals, White individuals) have more privilege than others (e.g., women and people with non-conforming genders, LGBTQ individuals, people of color). If we take a look at our history, we will see that the definition of who is White and who is not has evolved over time. People of Eastern European, Irish, Italian, Jewish, and Greek descent were not always recognized as being White, but because there were material advantages associated with being White, these groups of people fought to be included in this racial category. That the definition of who gets to be White has changed is evidence that race is socially constructed.

2. What if a learner questions why we need to think about this? For example, one participant once asked C.J.C (who identifies as Asian American and Chinese American) when she was going to stop thinking of herself as Asian American and begin just identifying as American.

- **Where this is coming from**: Similar to the question above, a learner who is asking this question has likely grown up in a very homogeneous environment and thinks of themselves as colorblind, race-blind, gender-blind, etc. They have been taught that these differences do not matter and that thinking about or noticing or commenting on these differences is an indication that one is discriminatory (e.g., racist, sexist, etc.). The problem with this line of thinking is that we do, in fact, notice such differences, and pretending that these differences do not exist can be hurtful to an individual. Being from a certain racial/ethnic, religious, gender identity is a big part of who people are—for someone to say they don’t see it negates a large part of one’s life experience and identity, Refusing to recognize differences can also be harmful (e.g., physicians who refuse to acknowledge patients’ predispositions to certain diseases because they do not want to acknowledge differences can act in ways that harm patients).
- **Suggested response**: Acknowledge that it would be nice if we were at a point where we could just see each other as “people” or “humans” or “American” and not have to include additional descriptors. Explain that our society is still in a place where these differences matter (C.J.C. brought up personal experiences of discrimination as well as a reference to the 1998 “American beats out Kwan” Olympic headline <http://community.seattletimes.nwsource.com/archive/?date=19980303&slug=2737594> to explain that she does not refer to herself as “just” an American because American society won’t let her.) Affirm that noticing these differences is important and is not divisive.

3. What if a learner who is White but who is also part of another minoritized group (e.g., White but from a lower socioeconomic status, or White but Jewish), insists they do not have any privilege? (Also addressed in Appendix C)

- **Where this is coming from**: It is must easier to acknowledge our oppression than our privilege. We are much more aware of our oppression because we can point to instances and examples of being mistreated. It is hard to recognize our privilege because it is hard to be cognizant of why we are being treated well. It is also much easier to deny that we have any privilege than to own up to it.
- **Suggested response**: Acknowledge that identities are intersectional and that most of us identify with identities from both privileged and oppressed groups. It is important to acknowledge all facets of our identities and recognize that while in some spaces we are oppressed, in others, we have privilege, and that it is important to use our privileged positions to speak out for those who do not have privilege.

Part 5: References and optional pre-reading for the facilitator

| **Reference** | **Summary** |
| --- | --- |
| DiAngelo, RJ. *What does it mean to be white?: Developing white racial literacy*. New York, NY: Peter Lang; 2016. (In particular, Chapter 3: “Socialization”) | Author uses Chapter 3 to discuss the concept of socialization and how identities are socially constructed. These social positions provide certain frames and lenses through which we see and make sense of the world. |
| Johnson, AG. *Privilege, power, and difference*. Boston, MA: McGraw-Hill; 2006. (In particular, Chapter 2: “Privilege, oppression, difference”) | Author uses Chapter 2 to walk through his own identities and explain how he came to understand how his identity, as a White, middle-class man, has influenced his world view. The author also provides a detailed list of the ways in which members of different privileged groups see the world differently from their less privileged counterparts. |
| Matias, CE. *Feeling White: Whiteness, emotionality, and education*. Rotterdam, The Netherlands; 2016. (In particular, Chapter 10: “Who you callin’ white?! A critical counter-story on colouring white identity”) | Author uses Chapter 10 to explicate how two former White teacher education students make sense of their Whiteness. The author critically dissects the students’ journal entries to reveal one student’s transformation toward understanding Whiteness and another student’s blatant refusal to recognize the existence of Whiteness and White supremacy. |
| Tatum, BD. *“Why are all the black kids sitting together in the cafeteria?”: And other conversations about race*. New York, NY: Basic Books; 1997. (In particular, Chapter 3: The early years) | Author uses Chapter 3 to explain how much children notice about difference during the early years, and how important it is to let children ask questions about difference. The author asserts that when we do not encourage children to talk about differences, or we discourage them from doing so, they learn that talking about differences in and of itself is bad. |
