# Supplementary material for: Tools for Discussing Identity and Privilege Among Medical Students, Trainees, and Faculty
Source: MedEdPORTAL. 2019 Dec 20;15:10864. doi: 10.15766/mep_2374-8265.10864 (PMC7012312; doi:10.15766/mep_2374-8265.10864)
Supplement: Supplementary file 1 — A. Identity Wheel Instructions.docx B. Identity Wheel Handouts.docx C. Group Reading.docx D. Marshmallow and Pretzel Activity.docx E. Survey.docx [file mep-15-10864-s001.zip › B. Identity Wheel Handouts.docx]

Appendix B: Identity Wheel

Instructions: Fill out the wheels below with your identity descriptors. Given identities are those that you are born with (e.g., race, ethnicity, sex, age). Chosen identities are those that you have chosen to identify with (e.g., gender, geographic location, religion). The wheel has been left blank so that you can choose with given and chosen identities to use.

Chosen

Given

Instructions: Fill out the wheels below with your identity descriptors. Given identities are those that you are born with (e.g., race, ethnicity, sex, age). Chosen identities are those that you have chosen to identify with (e.g., gender, geographic location, religion).

Hobbies

Geographic location

Career

Religion

Race

Sex

Sexual Orientation

Ability

Nationality

Age

Chosen

Given

Marital Status
