# Supplementary material for: Tools for Discussing Identity and Privilege Among Medical Students, Trainees, and Faculty
Source: MedEdPORTAL. 2019 Dec 20;15:10864. doi: 10.15766/mep_2374-8265.10864 (PMC7012312; doi:10.15766/mep_2374-8265.10864)
Supplement: Supplementary file 1 — A. Identity Wheel Instructions.docx B. Identity Wheel Handouts.docx C. Group Reading.docx D. Marshmallow and Pretzel Activity.docx E. Survey.docx [file mep-15-10864-s001.zip › C. Group Reading.docx]

**Appendix C: Group Reading**

Part 1: Primary reading

Clandinin J, Cave MT, Cave A. Narrative reflective practice in medical education for residents: composing shifting identities. *Adv Med Educ Pract.* 2011;2:1-7.

Part 2: Rationale/background

We use the Clandidin et al., article as a way to continue the conversation around developing a professional identity. We want participants (particularly when we are presenting to students and trainees) to understand that we do not just step into professional identities, but that the process of becoming a professional is ongoing and takes practice. This piece illustrates well how this process of development occurs. In addition, in order to build on the concepts learned in the identity wheel exercise, we want students to understand that taking on a professional identity involves negotiating how one’s personal and professional identities fit together. The Clandinin et al., piece is less explicit about this connection, so in the reference section (Part 6), we provide some optional readings.

Part 3: Reading (5-20 minutes)

1. Pass out article, have everyone open a digital copy, or project article on screen.
2. Read article aloud together. You can invite students to take turns reading excerpts, or appoint an individual to read the entire piece. (We found that reading together, rather than silently, was an effective use of the session time because we could interject comments as we are reading.)
3. If you do not have time to read the article in full, we suggest reading the excerpt “Table 1: Leslie’s Final Parallel Chart” as it offers a concise summary of the article’s main points.

Part 4: Discussion (15-30 minutes)

When we have a large group, we ask learners to do a pair-share, or talk in groups of three or four before sharing as a large group. Engage learners in a discussion using the following guiding questions:

1. What can we learn from Leslie’s experience?
2. What does it mean to be a bad doctor? A good doctor?
3. How do you view yourself as a physician (or as a future physician)?
4. How do you want to be viewed by patients?
5. What are experiences, both personal and professional, that have shaped your professional identity?
6. Closing:
   1. Ask learners to think of three things they would like to see in themselves by the end of medical school/training/in the next five years (depending on the audience).
   2. Ask learners to write these three things down as a way to formally reflect on the idea of professional identity (we do not usually have them share the answers with us).

Part 5: Potential challenges

It is not unusual for learners to be resistant when asked to talk about issues of identity and privilege. Below we provide examples of some of the questions we have received and how we have responded.

1. A learner asks why physicians (or professionals) cannot just see themselves with respect to their professional roles, e.g., “When are women physicians just going to see themselves as physicians? Why do they need to remind everyone that they’re women? Why can’t they just be physicians?

- **Where this is coming from**: Students who ask this question often identify with a majority group (e.g., white, male, middle- to upper-middle-class), and will likely have been socialized to believe that their social identities have no bearing on their professional identities or professional work because their social identities have never been explicitly discussed. It is easy for them to think of themselves as “just” a doctor because their social identities have never given anyone reason to question their ability to be a doctor. Finally, because they have been part of the majority, they have been socialized to think that they are “normal” and “human”, rather than individuals who have been shaped by social forces. This is the main reason we suggested doing the identity wheel exercise (Appendices A and B) first, so students can begin to think about the social forces at work in identity construction.
- **Suggested response**: Acknowledge that it is interesting that some people choose to just identify with their professional identities, while others find it important to name how their social and professional identities intersect. Ask why this might be (and potentially invite other students to respond). Explain that identities are socially constructed and that individuals who belong to minoritized groups (e.g., women, people of color, individuals from lower socioeconomic statues, individuals who identify with non-binary genders) often feel the weight of those identities much more than individuals who belong to non-minoritized groups because they know what it is like to feel different all the time, or almost all the time. Because these experiences have influenced these individuals’ lives and self-conceptions, they are often more conscious of how these social identities influence every aspect of life, including their professional lives. In addition, they are likely to see how the personal and professional overlap, which is why they see themselves as both and, rather than either/or.

Part 6: References and optional pre-reading for the facilitator

| **Reference** | **Summary** |
| --- | --- |
| Chow CJ, Byington CL, Olson LM, Ramirez KPG, Zeng S, Lopez AM. A conceptual model for understanding academic physicians’ performances of identity: Findings from the University of Utah. Acad Med. 2018;93:1539-1549. | Qualitative study that examines how physicians think about professional identities with respect to how they intersect with social identities (e.g., race, gender, class). |
| Cope A, Bezemer J, Mavroveli S, Kneebone R. What attitudes and values are incorporated into self as part of professional identity construction when becoming a surgeon? Acad Med. 2017;92:544–549 | Qualitative study that examines how surgeons have used personal values to inform their professional work and identities as surgeons. |
| Cruess RL, Cruess SR, Boudreau JD, Snell L, Steinert Y. A schematic representation of the professional identity formation and socialization of medical students and residents: A guide for medical educators. Acad Med. 2015;90:718–725. | Conceptual article that examines how professional identity formation takes place. Authors posit that individuals learn to connect their personal identities with their professional identities through socialization in the profession. |
| Goldie J. The formation of professional identity in medical students: Considerations for educators. Med Teach. 2012;34:e641–e648. | Article presents a conceptual model which utilizes principles of social psychology to illustrate how professional identity formation takes place. |
| Wald HS. Professional identity (trans)formation in medical education: Reflection, relationship, resilience. Acad Med. 2015;90:701–706. | Conceptual article that examines process of and factors influencing professional identity formation. Factors include progress in professional training and personal experiences. |
| Tweedy, D. *Black Man in a white coat: A doctor’s reflections on race and medicine.* New York, NY: Picador; 2015. | The author recounts his experiences of being a Black medical student, trainee, and now physician and the stereotypes and racism he’s encountered. The author interweaves data on social determinants of health with his personal account. |
