# Supplementary material for: Tools for Discussing Identity and Privilege Among Medical Students, Trainees, and Faculty
Source: MedEdPORTAL. 2019 Dec 20;15:10864. doi: 10.15766/mep_2374-8265.10864 (PMC7012312; doi:10.15766/mep_2374-8265.10864)
Supplement: Supplementary file 1 — A. Identity Wheel Instructions.docx B. Identity Wheel Handouts.docx C. Group Reading.docx D. Marshmallow and Pretzel Activity.docx E. Survey.docx [file mep-15-10864-s001.zip › D. Marshmallow and Pretzel Activity.docx]

**Appendix D:** The Marshmallow and Pretzel Exercise

Part 1: Rationale/background

This exercise has been used in teacher education and higher education programs and was introduced to us by the third author (C.E.M.). While this exercise can be done using different sized sticks, we have adapted the exercise to include the use of different sized pretzels.

The exercise is designed to build on the self-awareness activities that have just been completed, and move into understanding privilege in action, structural and historical inequity, and how leveling the playing field does not adequately solve issues of inequity. The goal of this exercise is to have the participants see how their lives and actions are influenced by and enmeshed within systems of privilege.

Part 2: Exercise #1­ (5 minutes)

Materials: Set up bags prior to the session. Pretzels and marshmallows should be different sizes—some groups will get small sticks, others will get full-length rods, some get mini marshmallows, others get regular marshmallows. You can also use toothpick and skewers instead of differently sized pretzels. If you want to further emphasize how disparities are reinforced, and how blind we often are to our privileged positions, you can make a bag with *only* marshmallows or pretzels—which makes it nearly impossible to build any structure. (We usually put materials in numbered brown paper bags. The numbers help us differentiate between the bags, but hide the varying materials from the groups.) We have found it useful to put the marshmallows in a zip-top bag inside the paper bag to reduce stickiness and staleness.

This exercise is designed to get students thinking about inequality and disparities i.e., how resources are unequally distributed. This also provides an opportunity for students to reflect on who usually notices that inequalities even exist.

1. Divide participants into groups of 2 to 4, depending on the size of the group, and give each group pretzel sticks and marshmallows.
2. Ask participants to build the tallest tower possible and give them 3 minutes to do so.
3. After the 3 minutes is up, congratulate the group with the tallest tower.


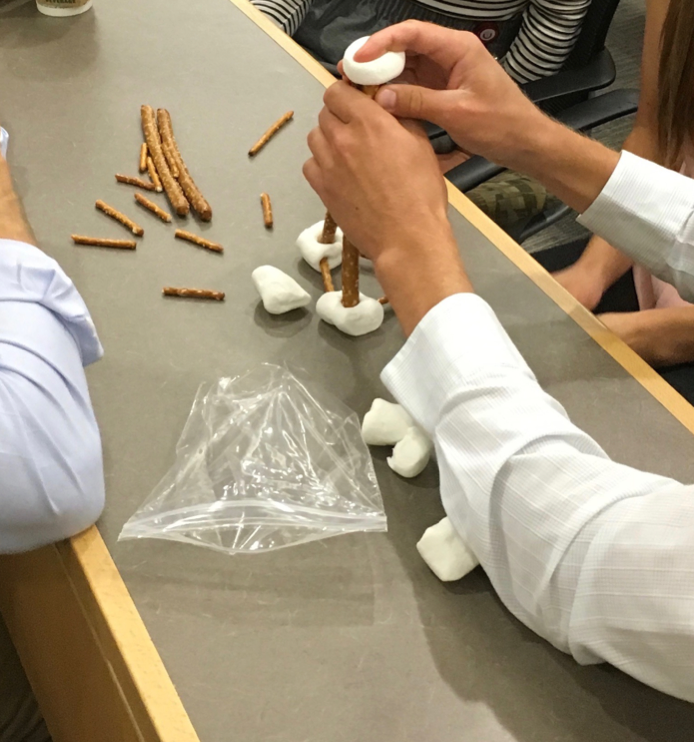


Author owned

Part 3: Discussion Part A (5-10 minutes)

At this point, we ask the groups to look around the room and see what they notice. Then, we use the following guiding questions to lead a discussion:

1. Look around the room. What do you notice?
2. Who has more resources? Who has less?
3. Who noticed that different groups had different resources? (It is not unusual for this to be the first time that groups notice that every group has a different set of materials, and to talk about how they were so caught up in their task that they did not notice that others had less or more. If you gave one group only marshmallows or pretzels, this is a good time to point out that the only group who was very aware of what they had in comparison to others is the group that had less).
4. Discuss how it was a privilege for the other groups not to notice that one group did not have adequate resources, and that we often do not notice our privileged identities because we do not have to.

Part 4: Exercise #2 (5 minutes)

Materials: Set up bags prior to the session. Bags should contain equal numbers of pretzels and marshmallows and pretzels should be all the same size. No need to hide the zip-top bags in paper bags this time since all bags have identical materials.

1. Acknowledge that what you just did was an unfair process, and tell participants that you have decided to level the playing field.
2. Give everyone new materials: bags with the same number and size of marshmallows and pretzels.
3. Tell groups they may start building. The one catch is that they can build on their existing structure.
4. Stop groups after 3 minutes.

Part 5: Discussion Part B (15-25 minutes)

We bring the group back together and ask them to reflect on this activity, and to share their thoughts and feelings about the process. Participants usually have a lot to say. Below are some guiding questions we have used:

1. Have you ever done an activity like this before? If so, what was that activity, and how do they compare?
2. Why isn’t “leveling the playing field” by giving everyone equal resources an effective way to address a history of inequality?
3. How has history produced an unequal foundation?
4. Why is it important to think about privilege as an ongoing issue/process, rather than just as isolated incidents?
5. Which identities (from the identity wheel/profile) give you privilege?
6. How does this exercise help you understand the historical nature of issues including racism, classism, sexism, and heterosexism?
7. What does this activity teach you about teamwork and sharing knowledge/roles within an inter-professional community, when you are (or will be) the physician, to whom power is usually attributed?
8. Closing
9. Talk about how all of these activities fit together: how self-awareness and recognition of identity privilege is a first step towards making healthcare more inclusive. We discuss how being a physician is a privilege in itself, despite what racial, socioeconomic, gender, religious background individuals may hold. Being a physician comes with a tremendous amount of social privilege and professional privilege, and it is important to understand how to use this privilege for good—to help others, to learn about health disparities, and to address inequalities when possible.
10. Ask participants to write down one to three ways they can be more conscious about their identities (and identity privilege) in their practice. This can include seeking out further professional development opportunities, finding ways to make more connections with patients from different backgrounds, etc.

Part 6: Potential challenges

1. What if a learner who is White but who is also part of another minoritized group (e.g., White but from a lower socioeconomic status, or White but Jewish), insists they do not have any privilege because of their minoritized identity? (Also addressed in Appendix A)
   - **Where this is coming from**: It is must easier to acknowledge our oppression than our privilege. We are much more aware of our oppression because we can point to instances and examples of being mistreated. It is hard to acknowledge our privilege because it is hard to be cognizant of why we are being treated well. It is also much easier to deny that we have any privilege than to own up to it.
   - **Suggested response**: Acknowledge that identities are intersectional and that most of us identify with identities from both privileged and oppressed groups. It is important to acknowledge all facets of our identities and recognize that while in some spaces we are oppressed, in others, we have privilege, and that it is important to use our privileged positions to speak out for those who do not have privilege.
2. What if a learner is generally resistant to the idea that privilege exists?
   - **Where this is coming from**: Recognizing privilege, particularly Whiteness, is difficult. As DiAngelo^2^ says, White individuals are taught from a very early age that being racist is bad and not being racist is good. Therefore, being told or acknowledging that one is racist is akin to acknowledging that one is a terrible person.
   - **Suggested response**: Explain that this good/bad binary is false, and that it is possible to be a good and caring person and also have discriminatory beliefs. Acknowledging racism and discrimination in one’s life, as well as privilege, is not about asking those with privilege to feel guilty. It is about understanding that resources are inequitably distributed and being committed to righting historical wrongs. Also acknowledge that lessons like these take a long time to learn, and that if a learner is not ready to accept this yet, that’s okay.
3. Chapter 14 from DiAngelo’s^2^ book includes a number of potentially difficult questions and responses. We highly recommend reviewing Chapter 14 for additional issues and ways to address them

Part 7: References and optional pre-reading for the facilitator

| **Reference** | **Summary** |
| --- | --- |
| DiAngelo, RJ. *What does it mean to be white?: Developing white racial literacy*. New York, NY: Peter Lang; 2016. (In particular, Chapter 4: “Defining Terms” and Chapter 14: “Popular white narratives that deny racism”) | Author uses Chapter 4 to define key terms including prejudice, discrimination, and racism. In Chapter 14, the author poses several hypothetical questions that individuals may have about race and racism and provides an explanation of how to respond to these questions. |
| Johnson, AG. *Privilege, power, and difference*. Boston, MA: McGraw-Hill; 2006. (In particular, Chapter 2: “Privilege, oppression, difference” and Chapter 4: “Making Privilege Happen) | Author uses Chapter 2 to walk through his own identities and explain how he came to understand how his identity, as a White, middle-class man, has influenced his worldview. The author also provides a detailed list of the ways in which members of different privileged groups see the world differently from their less privileged counterparts. In Chapter 4, the author explores how privilege persists. |
