# Supplementary material for: Tools for Discussing Identity and Privilege Among Medical Students, Trainees, and Faculty
Source: MedEdPORTAL. 2019 Dec 20;15:10864. doi: 10.15766/mep_2374-8265.10864 (PMC7012312; doi:10.15766/mep_2374-8265.10864)
Supplement: Supplementary file 1 — A. Identity Wheel Instructions.docx B. Identity Wheel Handouts.docx C. Group Reading.docx D. Marshmallow and Pretzel Activity.docx E. Survey.docx [file mep-15-10864-s001.zip › E. Survey.docx]

**Pre-Survey**

The purpose of this research study is to explore how individuals discuss their identities and their perceptions of culturally responsive and inclusive patient care/teaching. We are doing this study because we want to provide individuals an opportunity to explore social and professional identities and hope to improve approaches to future teaching on this subject.

We are asking you to complete a survey at the beginning of this session and second survey at the end of the session. Surveys and classroom discussions during the session may involve sharing information about social and professional identities that is considered personal or sensitive. Potential benefits of the research are that you will gain more knowledge of your professional and personal identities and that future teaching on this subject will be improved.

This survey is anonymous, and all survey data will be presented in aggregate.

Contact the Institutional Review Board (IRB) if you have questions regarding your rights as a research participant. Also, contact the IRB if you have questions, complaints, or concerns which you do not feel you can discuss with the investigator.

It should take 10 to 15 minutes to complete each questionnaire. Participation in this study is voluntary. You can choose not to take part. You can choose not to finish the questionnaire or omit any question you prefer not to answer without penalty or loss of benefits.

By returning this questionnaire, you are giving your consent to participate. We are grateful for your participation in this study.

We are all members of different social groups or social categories. Some of such social groups or categories pertain to gender, race, religion, nationality, ethnicity, and socioeconomic class. We would like you to consider your membership in those particular groups or categories, and respond to the following statements on the basis of how you feel about those groups and your memberships in them. There are no right or wrong answers to any of these statements; we are interested in your honest reactions and opinions. Please read each statement carefully, and respond (Luhtanen & Crocker, 1992).

| 1. Overall, my group memberships have very little to do with how I feel about myself. | Strongly disagree  1 | 2 | 3 | 4 | 5 | 6 | Strongly Agree  7 |
| --- | --- | --- | --- | --- | --- | --- | --- |
| 2. The social groups I belong to are an important reflection of who I am. |  |  |  |  |  |  |  |
| 3. The social groups I belong to are unimportant to my sense of what kind of person I am. |  |  |  |  |  |  |  |
| 4. In general, belonging to social groups is an important part of my self-image. |  |  |  |  |  |  |  |
| 5. The social groups I belong to have influenced my decision to become a physician. |  |  |  |  |  |  |  |
| 6. Overall, my group memberships have very little to do with how I feel about practicing medicine. |  |  |  |  |  |  |  |

**Post-Survey**

The purpose of this research study is to explore how individuals discuss their identities and their perceptions of culturally responsive and inclusive patient care/teaching. We are doing this study because we want to provide individuals an opportunity to explore social and professional identities and hope to improve approaches to future teaching on this subject.

We are asking you to complete a survey at the beginning of this session and second survey at the end of the session. Surveys and classroom discussions during the session may involve sharing information about social and professional identities that is considered personal or sensitive. Potential benefits of the research are that you will gain more knowledge of your professional and personal identities and that future teaching on this subject will be improved.

This survey is anonymous, and all survey data will be presented in aggregate.

Contact the Institutional Review Board (IRB) if you have questions regarding your rights as a research participant. Also, contact the IRB if you have questions, complaints, or concerns which you do not feel you can discuss with the investigator.

It should take 10 to 15 minutes to complete each questionnaire. Participation in this study is voluntary. You can choose not to take part. You can choose not to finish the questionnaire or omit any question you prefer not to answer without penalty or loss of benefits.

By returning this questionnaire, you are giving your consent to participate. We are grateful for your participation in this study.

We are all members of different social groups or social categories. Some of such social groups or categories pertain to gender, race, religion, nationality, ethnicity, and socioeconomic class. We would like you to consider your membership in those particular groups or categories, and respond to the following statements on the basis of how you feel about those groups and your memberships in them. There are no right or wrong answers to any of these statements; we are interested in your honest reactions and opinions. Please read each statement carefully, and respond (Luhtanen & Crocker, 1992).

| 1. Overall, my group memberships have very little to do with how I feel about myself. | Strongly disagree  1 | 2 | 3 | 4 | 5 | 6 | Strongly Agree  7 |
| --- | --- | --- | --- | --- | --- | --- | --- |
| 2. The social groups I belong to are an important reflection of who I am. |  |  |  |  |  |  |  |
| 3. The social groups I belong to are unimportant to my sense of what kind of person I am. |  |  |  |  |  |  |  |
| 4. In general, belonging to social groups is an important part of my self-image. |  |  |  |  |  |  |  |
| 5. The social groups I belong to have influenced my decision to become a physician. |  |  |  |  |  |  |  |
| 6. Overall, my group memberships have very little to do with how I feel about practicing medicine. |  |  |  |  |  |  |  |

7. Thinking back over today’s session, was there any particular concept that resonated with you? Did you have an “ah-ha” moment? What was it about?

8. What new approaches to thinking about your social identities will you take away?

9. What additional resources do you need to continue learning about these issues?

10. What is one way you plan to make your interactions with future patients more inclusive?

1. Additional comments/feedback for facilitators:
